# A Concise Review of State-of-the-Art Sensing Technologies for Bridge Structural Health Monitoring

**DOI:** 10.3390/s25175460

**Published:** 2025-09-03

**Authors:** Xiushan Kang, Bing Zhu, Yougang Cai, Yufeng Xiao, Ningbo Liu, Zhongxu Guo, Qi-Ang Wang, Yang Luo

**Affiliations:** 1 School of Civil Engineering, Southwest Jiaotong University, Chengdu 610031, China; 2 China Construction Fourth Engineering Division Corp. Ltd., Chengdu 610299, China; 3 School of Mechanics and Civil Engineering, China University of Mining and Technology, Xuzhou 221116, China

**Keywords:** structure health monitoring, RFID, carbon nanotube technology, piezoelectric sensing technology, optical fiber sensing technology

## Abstract

Against the backdrop of increasing demands for the safety and longevity of the bridge infrastructure, this review synthesizes the recent advances in structural health monitoring (SHM) sensing systems. Carbon nanotube (CNT), piezoelectric, RFID, wireless, fiber optic, and computer-vision-based sensing are thoroughly explored and elucidated in the existing literature survey that distills their working principles, documented deployments, and anticipated research directions. CNT sensors detect minute resistance variations for strain and crack surveillance; piezoelectric devices transduce mechanical stimuli into high-resolution electrical signals; RFID tags combine location tracking with modular sensing and wireless data relay; and wireless sensing technology integrates sensor nodes with microprocessors and communication modules, which can facilitate efficient data processing and autonomous management. Fiber optic sensing technology, known for precision and interference resistance, is ideal for high-precision monitoring under strong electromagnetic interference conditions, and vision-based systems emulate human perception to extract geometric descriptors via image analytics. The comparative analysis reveals complementary strengths that guide practitioners in selecting optimal sensor suites for specific bridge conditions. The findings underscore the transformative role of these technologies in enhancing SHM reliability and suggest that synergistic integration with robotics and emerging materials will further advance future resilient monitoring frameworks.

## 1. Introduction

In the domain of bridge structural health monitoring (SHM), advanced sensing technology has become an essential tool for evaluating structural performance and ensuring safe operation [1,2,3]. Sensing technologies facilitate real-time monitoring, long-term response data collection, and the assessment of structural safety, enabling a proactive approach for identifying structural damage and implementing timely maintenance measures. The integration of advanced sensing devices empowers bridge managers with accurate insights into the structural condition, leading to enhanced safety, improved structural durability, and a prolonged lifespan [4,5,6]. According to statistics, the coverage rate of the bridge structure health monitoring system is not very high, and the SHM system is generally used in some extra-long-span bridges. In the SHM system of the extra-long-span bridges, the acceleration, strain, displacement, temperature and humidity, and cable force are often physical quantities that are generally measured, and up to 300–500 sensors can be deployed on a single bridge. Statistics show that crack propagation, cable force deviation, and fatigue damage account for a large proportion of abnormal alarms, while the rest are bearing settlement, scouring, and other alarms. The average warning accuracy of the system is high, and the false alarms mainly come from sensor errors, external environmental interference, and abnormal data transmission.

Traditional sensors encompass well-established point-wise devices such as electrical strain gauges, linear variable differential transformers, thermocouples, and wired accelerometers; these systems have provided decades of reliable data but remain constrained by cabling, power, and limited spatial coverage. Advanced or emerging sensors, in contrast, leverage novel materials, energy-autonomous operation, and distributed intelligence, e.g., carbon-nanotube self-sensing concrete, chipless RFID sensor tags, batteryless wireless smart nodes, AI-driven computer-vision networks, etc. Advanced sensing technology in bridge structural health monitoring offers several significant advantages and applications [7,8,9,10]. It enables real-time monitoring, allowing for the continuous observation of crucial environmental parameters and structural responses, e.g., temperature, humidity, deformation, strain, stress, acceleration, etc. SHM sensors collect and transmit data in real time, providing bridge managers with prompt insights. The technology ensures high-precision data acquisition, capable of recording small deformations and stress variations, which is essential for accurate structural health assessment. Additionally, advanced sensing allows for multi-parameter monitoring, enabling the simultaneous observation of various responses to provide a comprehensive evaluation of the structural performance. This capability aids in identifying abnormal behaviours and potential structural damage. Data analysis and prediction are enhanced through the utilization of machine-learning algorithms, which process the collected data to predict the structure’s remaining life and future behaviour, thereby assisting in informed decision-making for timely maintenance and repair. Furthermore, the advanced sensing technology facilitates anomaly detection and alerts, identifying abnormal behaviours in bridge structures and issuing alerts based on pre-defined thresholds, which helps in taking preventive measures to avoid catastrophic failures. In addition, unmanned aerial vehicles now serve as agile, cost-effective platforms for drone-based bridge inspections, rapidly capturing high-resolution imagery and light detection and ranging data over large or inaccessible spans while minimizing traffic disruption. These aerial systems are increasingly equipped with lightweight micro-electro-mechanical-systems inertial measurement units, strain gauges, accelerometers, and environmental sensors that deliver real-time multi-parameter data streams. The fusion of micro-electro-mechanical-systems’ precision with unmanned aerial vehicle mobility enables simultaneous geometric mapping, vibration monitoring, and crack detection from the air, extending the traditional ground-based SHM to adaptive, large-scale screening campaigns.

Recent reviews of bridge structural health monitoring (SHM) generally focused on a single sensing technology, such as fiber Bragg gratings or vision-based crack detection. The investigation of emerging technologies such as carbon nanotube sensing, hybrid piezoelectric energy harvesters, and chipless RFID applications is not sufficient. The present review work is motivated by the need to close these gaps, and it systematically benchmarks the latest advances in CNT, piezoelectric, RFID, wireless, optical fiber, and computer-vision sensing against common performance metrics (sensitivity, power budget, installation cost, and long-term stability), and explicitly highlights how these advanced sensing technologies are redefining the state of the art in bridge SHM. This study delves into the literature review and summary for state-of-the-art sensing technologies, including the carbon nanotube technology (CNT), piezoelectric sensing technology, wireless sensing technology, optical fiber sensing technology, and computer-vision-based sensing technology, focusing on their contributions and applications in the SHM field over the past decade. These technologies open up new dimensions for monitoring physical quantities and provide comprehensive Internet of Things (IoT) solutions for infrastructure health. By combining laboratory benchmarks with comprehensive field validation, the review distills the current limitations such as power sustainability, data security, and durability in harsh environments, and charts a future path towards a self-calibrating, edge-smart sensing ecosystem capable of lifetime, maintenance-free bridge management.

(1) In the pursuit of effective SHM methods, carbon nanotube technology has emerged as a promising sensing method. The incorporation of CNT in concrete has revealed self-sensing capabilities, demonstrating changes in electrical resistance during loading and crack propagation, as well as irreversible resistance changes in the inelastic stage. This method offers an alternative to traditional techniques for structural health monitoring. The self-sensing properties of composites with CNT showcase the potential for the cost-effective and efficient monitoring of advanced concrete structures. Moreover, incorporating carbon nanotubes into cement-based matrix composites and applying them in reinforced concrete beams showcases the material’s piezoelectric properties, offering a self-perceiving capability to monitor changes in global stiffness through resistance variations [11,12,13,14].

(2) Piezoelectric sensing technology, which can transfer physical quantities into electrical signals via the piezoelectric effect, plays a pivotal role in the field of structural health monitoring. This study systematically reviews the significant advancements and applications of this technology. These studies focus on the development of novel piezoelectric sensor designs, underscoring the ongoing evolution and refinement of this critical technology in SHM.

(3) Wireless sensor technology represents a sophisticated network system that interconnects multiple sensor nodes via wireless communication pathways. In the field of bridge structural health monitoring, this technology offers significant advantages by facilitating real-time data collection and analysis, unencumbered by the limitations imposed by wired connections. Wireless sensors are adept at functioning in challenging environments, support distributed computing frameworks, and offer cost-efficient solutions for the SHM of large-scale structures. Their utility extends beyond structural monitoring; for instance, they are employed in the detection of metal surface defects and the monitoring of cement mortar hydration processes, underscoring their versatility and efficacy across a wide range of applications.

(4) Optical fiber sensing technology is a highly precise and reliable method for structural health monitoring, known for its strong anti-interference capabilities and ease of installation. It is widely applied in assessing structural integrity, measuring deformation, and monitoring thermal conditions. The technology utilizes fiber optic sensors to detect and analyze various physical changes within structures. Its applications extend to seismic detection, corrosion monitoring, traffic information systems, etc., providing comprehensive data for structural health monitoring. The advantages of optical fiber sensing include its ability to deliver detailed insights into structural behaviour, enabling the early detection of potential issues and informing timely maintenance decisions to ensure safety and longevity.

(5) The operational principle of computer-vision-based sensing technology is derived from biological vision, enabling machines to perceive the world in a human-like manner. By simulating biological visual mechanisms through visual sensors, this technology acquires characteristic parameters of target objects—including the area, centroid, length, and positional coordinates—and subsequently processes these visual inputs through advanced image analysis algorithms. The system ultimately outputs quantified data and decision outcomes, thereby serving as the direct source of information for computer-vision-based systems. This biometrically inspired approach establishes a fundamental framework for automated visual perception and intelligent decision-making in technological applications. This article aims to (1) systematically sort out and quantitatively compare the latest progress of five emerging sensing technologies, namely, intelligent sensing enabled by the carbon nanotube technology, piezoelectric sensing technology, wireless sensing technology, optical fiber sensing technology, and computer-vision-based sensing technology, in bridge structure health monitoring; (2) fill the gap in the literature review on bridge SHM over the recent past decade that lacks a comprehensive assessment of cross-technical performance indicators (sensitivity, power consumption, installation cost, and long-term stability); and (3) quantitatively compare laboratory and field deployments to reveal cross-technology advantages and limitations of advanced sensing technologies.

## 2. State-of-the-Art Sensing Technologies for Bridge SHM

As critical links in transportation networks, bridges are exposed to escalating traffic loads, aggressive environments, and ageing materials, which may cause catastrophic failures both costly and life-threatening. Therefore, SHM systems serve as early-warning sentinels that enable proactive maintenance, extend service life, and safeguard public safety. The bridge structure health monitoring system uses a high-density sensor network to obtain multi-dimensional data such as strain, vibration, and environment in real time, establishes a digital twin model, and predicts the degradation of bearing capacity, significantly reducing the risk of life, economy, and traffic disruption caused by collapse. The system defines damage as follows: “the structural response deviates from the design baseline and is irreversible”. The damage features, e.g., cracks, corrosion, and fatigue, can be determined and extracted from the monitoring data through machine-learning algorithms, and the threshold or trend criterion can be used to automatically identify the damage location, degree, and development stage. Precise maintenance decisions can be triggered based on hierarchical early warnings, thereby extending the safe service life of the bridge and optimizing the entire life cycle cost.

### 2.1. Intelligent Sensing Enabled by the Carbon Nanotube Technology

Carbon nanotubes can serve as highly sensitive sensors in structural health monitoring by monitoring changes in their electrical properties, and can be utilized to enhance multifunctional composite materials for improved structural performance [15]. Castañeda-Saldarriaga et al. [16] investigated the production and multipurpose experimental characterization of a cement-based matrix (CBM) composite featuring carbon nanotube inclusions, along with its integration into a representative structural element. A self-made sensor was used to successfully self-perceive the change in global stiffness through the change in resistance. This study dispersed 0.8 wt% carbon nanotubes into a Portland cement matrix, cast cubes with copper electrodes, cured them for 28 days, and embedded a matching parallelepiped sensor inside a reinforced concrete beam using dowels. During three-point bending tests, the sensor continuously measured the electrical resistance while the load increased.

Rao et al. [17] presented a systematic method to fabricate a sensor (intelligent cement sensor) using a cement matrix and carbon nanotube nano-inclusion. Different types of multi-walled nanotubes and different percentages of cement matrix by weight were considered, with an influence on the strain sensitivity of the sensor under cyclic compression load. It is found that the developed CNC machine can capture the natural frequency of the structure, which is sufficient for vibration-based structural health monitoring. Ye. et al. [18] developed an intelligent CFRP for crack opening displacement (COD) monitoring and delay by embedding distributed fiber optic sensors. A theoretical model of the COD explicit expression considering the elastic and elastic–plastic properties of adhesives is established. The distributed fiber embedded in intelligent carbon fiber cloth improves the COD-monitoring accuracy and sensitivity and has an anti-crack detection ability for reinforced structures. Sathyanarayanan et al. [19] found that the incorporation of small quantities of short carbon fibers into mortar and concrete has demonstrated self-sensing or intelligent behaviour. This method can provide an alternative to traditional techniques like strain gauges or fiber optics for structural health monitoring.

In addition, some scholars have conducted relevant research based on the use of novel materials in the domain of structural health assessment. Yan et al. [20] proposed an innovative technique for enhancing CFRP with self-sensing capabilities. The sensor is composed of two CFRP layers with a titania-filled epoxy dielectric layer in between, thereby forming a parallel-plate capacitor. The sensing functionality is realized through variations in the sensor’s capacitance induced by strain, providing an additional feature for structural health monitoring and management. Downey et al. [21] introduce a computationally efficient resistor mesh framework designed to detect, locate, and measure the damage in structures constructed from conductive cement composites. This method is experimentally confirmed on both non-reinforced and reinforced samples of nanocomposite cement paste. McAlorum et al. [22] introduce an operating method for spray-coating geopolymers, a category of self-sensing concrete repair materials, which can provide strain and temperature measurements of the underlying concrete substrate with resolutions of 1 με and 0.2 °C, respectively. This advancement could facilitate the broader adoption of innovative concrete health monitoring and repair systems in the future. Zhan [23] investigates the effects of different types and contents of carbon nanotubes on the mechanical and electrical properties of cement-based materials. The composite material equivalent circuit and electromagnetic parameter analysis were explored. Carbon nanotube sensing mainly faces problems such as the dispersion of electrical properties and large environmental interference. The dispersion of electrical properties can be reduced through surface modification and hierarchical purification. Encapsulation and algorithm compensation can be used to suppress environmental interference. In the future, intelligent sensing enabled by carbon nanotube technology may focus on investigating the stretchable carbon nanotube network with flexible electronic heterogeneity, and the zero-power self-diagnostic wireless carbon nanotube sensing node.

### 2.2. Piezoelectric Sensing Technology

Piezoelectric sensing technology utilizes the piezoelectric effect to convert physical quantities (such as pressure, temperature, acceleration, etc.) into electrical signals [24,25,26]. Piezoelectric materials have a special crystal structure and electrical properties. When piezoelectric materials are subjected to external forces or temperature changes, the asymmetry of the charge distribution will be generated, resulting in an electric potential difference, which can be measured and converted into an electrical signal. Wang et al. [24] proposed a piezoelectric–triboelectric-hybrid-generator-based autonomous wake-up sensing method to address the persistent energy consumption issue in wearable motion monitoring. This on-demand sensing system utilizes triboelectric signals as triggers while recording piezoelectric voltage amplitudes for angle quantification, effectively minimizing unnecessary data transmission. Izhar et al. [25] developed a novel piezoelectric-based energy harvester that converts three ambient energies (acoustic, vibration, and wind) simultaneously into electrical energy for the long-lasting operation of wireless sensor nodes. Jung et al. [26] presented a new failure detection strategy by employing a multifunctional piezoelectric material for vibration sensing and energy harvesting. Specifically, this study designed a simple power management circuit that saves power proportional to the piezoelectric voltage so that the piezoelectric patch with a higher strain can transmit more frequent wireless signals. Sun et al. [27] introduced a hardware platform for the real-time wireless health monitoring of structures utilizing compressive sensing. The experimental results show that this framework demonstrates excellent signal measurement performance, making it suitable for long-term wireless structural health monitoring applications. Feng et al. [28] provided a review and discussion of the progress of piezoelectric technology in the domain of civil SHM. In addition, they provided a detailed introduction to one of the proactive detection methods, the electromechanical impedance (EMI) technique. Finally, based on the detailed discussion, they highlighted the tremendous potential of piezoelectric technology in the realm of civil SHM. Peralta-Braz et al. [29] introduce a bi-objective optimization framework for the piezoelectric energy harvesting (PEH) design, highlighting the trade-off between energy harvesting efficiency and sensing accuracy. The numerical model is based on the Kirchhoff–Love plate theory and isogeometric analysis.

In addition, some scholars have carried out a lot of research on piezoelectric sensing technology. Jiao et al. [30] provided an overview of the SHM application of piezoelectric sensing technology. The fundamental mechanisms and principles underlying the piezoelectric effect, as well as the utilization of piezoelectric sensing techniques for structural detection, are explored, the responses are introduced, and the advantages and limitations of the current methods are discussed. The authors discussed the advantages and limitations of the current techniques. He et al. [31] surveyed the present state and future directions of bridge structure monitoring, with a focus on advanced structural health monitoring technologies, sensing data transmission, and analysis methods. They outlined an integrated structural health monitoring framework that begins with a systematic visual inspection to classify the surface damage. The seismic performance was evaluated through a spectral analysis using site-specific response spectra and demand–capacity ratios. Dynamic model updating was carried out based on monitoring data through the automated iterative adjustment of twenty-six physical parameters until the analytical and measured responses converge, thereby progressively refining structural identification and enabling economical maintenance scheduling. Liao et al. [32] introduced a new island–bridge packaged piezoelectric sensor for SHM in high-strain tests. The novel sensor’s island–bridge design, featuring a trapezoidal cross-section, significantly improves the piezoelectric layer’s ability to withstand high strain.

Piezoelectric sensing technology faces some major problems, e.g., temperature drift and sensitivity attenuation. High-stability quartz crystals can be employed to suppress the temperature drift. Long-term sensitivity can be maintained by using temperature compensation algorithms and selecting high-sensitivity materials. In the future, piezoelectric sensing may focus on developing flexible piezoelectric film to achieve wearable non-inductive monitoring, zero-power self-calibrating piezoelectric sensing techniques, etc.

### 2.3. Wireless Sensing Technology

Wireless sensing technology is crucial in structural health monitoring, covering various methods such as RFID, ZigBee, LoRa, NB-IoT, etc. Among them, RFID can realize the real-time distributed perception of cracks, rust, strain, and other parameters in bridge health monitoring with its advantages such as low cost, passive power consumption, batch deployment of labels, and long-distance group reading, and has excellent application prospects.

Radio frequency identification sensing system can be used to identify and track physical objects, and to monitor the location, movement, and status of objects in real time and provide critical data for Internet of Things devices [33,34,35]. The RFID system generally consists of a reader and one or more tags that can be attached to an object. Liu et al. [36] introduced a microstrip-coupled RFID sensor without a chip that supports a 4-bit ID code and integrates temperature and humidity sensing capabilities. By applying linear normalization fitting, the sensitivity of the sensor in relative humidity testing is approximately 2.18 MHz/RH. They fabricated a chipless microstrip-coupled radio frequency identification sensor containing complementary-split-ring-and-electric-field-coupled resonators on a Rogers substrate. Polyvinyl alcohol and reduced graphene oxide films were coated on designated resonators to convert humidity and temperature into dielectric changes. The sensor was placed in a sealed box where the humidity and temperature were varying. Resonant frequency shifts were recorded by a vector network analyzer and linearly fitted, yielding the humidity sensitivity and temperature sensitivity of the designed sensor.

Sunny et al. [37] proposed an economical and efficient self-compensation method by selecting and fusing temperature-related features near the resonance region of the tag for self-scanned frequency measurements. Experimental tests confirmed the success of the approach in temperature compensation, and various initial findings demonstrated the capability of the technology in addressing non-uniformity. Wang et al. [10] tackle challenges such as extensive cabling and the need for a continuous power supply to address the limitations of conventional strain sensors. They introduce a novel dual-interrogation mode radio frequency identification strain sensor that extends the interrogation transmission distance for wireless strain sensing. The proposed design includes an RFID tag and reader for wireless strain transmission, along with an improved Wheatstone bridge for strain measurement.

In addition, Chen et al. [38] conducted an experimental analysis on corrosion-monitoring intelligent RFID systems and structural-health-monitoring experimental systems. The sample group for the intelligent RFID corrosion-monitoring system experiment includes coated and uncoated mild steel plates, with patches exposed to the environment for varying durations, resulting in varying degrees of corrosion. The experimental results confirm the method’s effectiveness and robustness. Dey et al. [39] introduced a chipless RFID-based universal crack-sensing method for structural health monitoring. The proposed plan entails the development of an innovative “smart skin” sensor capable of offering either the continuous or discrete monitoring of structural deformations across any location on its surface. The growth and expansion of cracks in building structures can be identified by the sensor. Meng et al. [40] suggest integrating wireless powering, sensing, and communication, proposing RFID and sensing (RFID&S) techniques to enhance data collection efficiency and integration for Industrial Internet of Things (IIoT) innovations.

In addition, wireless sensor technology utilizes wireless communication methods to connect multiple sensors to form a sensing network [41,42,43]. The technology includes a plurality of sensor nodes distributed in different locations, which are connected through wireless communication technology, and can realize the acquisition, transmission, and processing of the environment, objects, or human body and other perceptual information. Loubet et al. [44] studied the comparable implementation of batteryless sensing nodes in networked physical systems, discussing the concepts of wireless power supply and batteryless wireless sensors for information-physical systems specifically designed for structural health monitoring applications in harsh environments. The intelligent mesh wireless sensor network, consisting of sensing and communication nodes, forms the foundation of the proposed material architecture. Ying et al. [45] proposed a novel intelligent wireless sensor network for monitoring the health of large-scale structures, introducing the software architecture of the unit and demonstrating the distributed computing capability of the intelligent sensing network. The results indicate that this new intelligent wireless sensor network serves as an effective tool for monitoring the health of large-scale structures. Fu et al. [46] developed a smart wireless monitoring system as an affordable solution. This system uses ultra-low power, event-triggered wireless sensor prototypes to enable on-demand, high-fidelity sensing without missing unpredictable impact events. The system can pinpoint the collision site and quantify the maximum force. The acquired collision data facilitates the prompt assessment of structural integrity, allowing engineers to efficiently prioritize inspections of critically affected areas.

In addition, Li et al. [47] investigated an improved sensor for detecting metal surface defects based on a three-dimensional RFID tag antenna. They introduced a wireless passive 3D sensing antenna and confirmed its practicality through simulations. The results indicate that the antenna can effectively characterize the depth and width of smooth defects on metal surfaces in two extended directions. Chen et al. [48] investigated the antenna sensors’ response characteristics for detecting defects in metal structures. This research is of significant importance for the online assessment of the metal lifecycle in the SHM service field. Passive antenna sensors play a significant role in evaluating the metal lifecycle. Cui et al. [49] proposed an adaptive edge intelligence strategy to facilitate the autonomous structural condition assessment, incorporating a reference-free displacement estimation algorithm, Gaussian process regression, and stochastic process control. The strategy was validated using Xnode, a MEMS-based wireless sensor platform, through laboratory tests and full-scale implementations in railroad bridge monitoring. The results demonstrate the potential and suitability of the developed approach for rapid adaptive structural condition assessment in SHM practice.

The wireless sensing technology mainly faces the problems of metal interference and energy limitation. Anti-interference antenna design and signal processing can be used to reduce the influence of metals. By integrating energy-harvesting techniques, long-term wireless sensing without a battery can be realized. In the future, the main research directions of wireless sensing may focus on energy self-supply methods, and chipless RFID techniques, as well as high-efficiency data transmission techniques.

### 2.4. Optical Fiber Sensing Technology

Fiber optic sensing technology is a kind of structure monitoring technology based on the physical characteristics of optical fiber [50,51,52,53,54,55]. It offers high precision, strong anti-interference capabilities, and easy installation. At present, photonic detection systems have found broad application in the structural assessment of spans, enabling the measurement of deformation and thermal conditions.

Ou et al. [56] investigated many intelligent sensing technologies for monitoring the health of civil infrastructures. They presented an array of photonic detection technologies, encompassing diverse light-based grating sensors, composite-integrated optical detectors, intelligent cabling with embedded photonic elements, and dynamic mass measurement systems utilizing fiber optics. In addition, polymer-based deformation meters, smart material displacement detectors, cement-composite strain monitors, wireless motion and stress measurement networks, and satellite-based positioning systems were also investigated. Gong et al. [57] investigated an intelligent system utilizing a fiber Bragg grating and an erbium-doped fiber amplifier, which provides notable benefits and enhanced performance for ultrasound sensing applications. Soman et al. [58] developed a damage detection technique specially designed for fiber grating sensor networks. An AS network design method for improving the damage assessment ability is proposed. Two steps for the actuator layout optimization of the AS network based on the FBG sensor were proposed. Firstly, they calculated the necessary number of piezoelectric actuators from the sensor density theory that balances the damage reflection strength, signal-to-noise ratio, and temperature drift. A single fiber grating sensor was fixed at the plate centre and oriented along the *x*-axis. Secondly, an evolutionary computation algorithm optimized actuator positions by maximizing the plate coverage through direct actuator–sensor paths and edge reflections. The cost function weighted the coverage by at least two actuator–sensor pairs and coverage by edge reflections. Laboratory tests with added masses validated that the optimized layout accurately detects and localizes damage.

Wang et al. [59] engineered an advanced seismic detection setup employing optical fiber technology and laser-based signal processing. The sensitivity of the microseismic FBG sensor depends on the sensor shell structure and the shape of the FBG reflectivity spectrum. Experiments were conducted to assess the frequency characteristics of the sensor system. Zhan et al. [60] present the principles of FBG sensors and outline three key demodulation techniques: tunable Fabri–Perrot (F–P) filter demodulation, high-speed demodulation using a tunable laser with sweep wavelength, and linear filter demodulation with matched grating. They review the advancements in FBG-based monitoring technology for three moulding methods: hot compression moulding, fused deposition moulding, and automated fiber placement moulding.

In addition, Fan et al. [61] conducted a comprehensive review of various fiber optic sensor types utilized for corrosion monitoring within reinforced concrete structures. The types of sensors examined encompass interferometric sensors, reflectometer sensors, distributed sensors, and grating sensors. For every sensor type, the review encompasses the principles of sensing and the essential characteristics of their application, with an emphasis on elucidating the capabilities and performance of the sensors across various corrosion phases. Mustapha et al. [62] introduced a framework designed for the concurrent monitoring of crowds and structures, integrating perception technology with machine-learning processes to facilitate intelligent decision-making in crowd management. They present the results from measurements obtained from a prototype experimental testbed, demonstrating the effectiveness of the proposed framework and offering preliminary insights for future research extensions. Dong et al. [63] collected data from distributed fiber optic sensors embedded in a test road using an optical frequency domain reflectometer (OFDR). Through the analysis and processing of the gathered data, a traffic information monitoring approach utilizing a peak recognition algorithm was developed. An analysis of real-world traffic data demonstrated that distributed fiber optic sensors effectively monitor various vehicle types externally and achieve the precise identification of traffic flow, vehicle speed, and load positions.

Fiber optic sensing faces issues such as environmental interference, long-range attenuation, and fragile installations. Long-distance attenuation limits the monitoring range and can extend the distance with low-loss fibers through distributed amplification. In the future, optical fiber sensing may break through in some major directions, e.g., the ultra-high-density distributed network with a mixed multiplexing of space and wavelength division to achieve kilometer-level millimeter spatial resolution, flexible implantable optical fiber micro–nano structures for real-time physiological monitoring, etc.

### 2.5. Computer-Vision-Based Sensing Technology

In recent years, computer vision technology has made remarkable progress in the field of bridge structural health monitoring, covering many aspects such as structure identification, damage detection, load identification, and displacement measurement, providing an efficient, non-contact, and automated solution for bridge safety assessment and maintenance [64,65,66,67,68,69]. The existing research can be roughly divided into three categories: (1) structure identification and displacement monitoring: computer-vision-based sensing technology can be employed to identify the overall structural state of the bridge and monitor the dynamic displacement under load; (2) damage detection and assessment: image processing technology and deep learning can be used to automatically detect cracks, spalling, rust, and other damage and conduct a quantitative analysis; (3) load identification and load distribution analysis: computer-vision-based sensing technology can be used to monitor the vehicle flow, weight distribution, and dynamic load effect on the bridge to optimize the bridge design and maintenance strategies. With the combination of computer vision and deep learning, UAV inspection, and multi-sensor fusion technology, bridge health monitoring is developing towards a higher precision, stronger robustness, and wider applicability, providing strong technical support for the long-term safe operation of bridges.

In terms of structure identification and displacement monitoring, Khuc et al. [70] proposed a new structure identification framework (ST-ID), which combined vehicle load modeling and the image damage index to carry out a bridge state assessment, and verified it on laboratory bridge models. In addition, Lee and Shinozuka [71] developed a vision-based remote measurement system capable of the real-time monitoring of bridge dynamic displacement. Xu et al. [72] proposed a low-cost, non-contact multi-point displacement measurement system and verified it on cable-stayed bridges. Santos et al. [73] further discussed the visual measurement problem of long-span suspension bridges and proposed a full-motion estimation algorithm based on adaptive calibration.

In terms of damage detection and assessment, Yeum and Dyke [74] proposed an automated crack detection method, which combined computer vision technology and an image-processing algorithm to effectively detect cracks on the bridge surface. Liu et al. [75] proposed a bridge damage detection framework based on hierarchical semantic segmentation. Compared with traditional methods, the framework improved the performance of the structural components and damage segmentation tasks by 5% and 18%, respectively. Zhu et al. [76] combined transfer learning and convolutional neural networks (CNNs) to achieve efficient bridge defect detection and improve the accuracy and efficiency of detection.

In addition, Qiao et al. [77] proposed a Computer-Vision-Based Bridge Damage Detection method employing Deep Convolutional Networks with an Expectation Maximum Attention Module. They built a bridge damage dataset with 4300 pixel-level images of cracks and exposed steel bars captured by a camera on an inspection vehicle in Zhejiang province. They trained a modified densely connected convolutional network that replaced the original classifier with an upsampling path and an expectation-maximization attention module inserted after the last pooling layer to emphasize damage features. A connectivity-aware loss function was added to improve crack continuity. The network was trained on 80 percent of the data and validated on the remaining 20 percent, achieving a 79.87 percent mean intersection over union on the challenging bridge dataset and demonstrating robust detection under complex backgrounds. The method redesigned the structure of densely connected convolutional networks (DenseNet) and incorporates an Expected Maximum Attention (EMA) module after the final pooling layer to improve bridge damage feature extraction. Additionally, a new connectivity-aware loss function was introduced, which effectively reduces breakpoints in fracture predictions and enhances accuracy by considering pixel connectivity.

In terms of load recognition and load distribution analysis, Zhou et al. [78] proposed a vehicle load recognition method based on non-contact machine vision and a deep-learning algorithm, in which the Faster R-CNN can effectively detect the vehicle position and calculate the load combined with the post-processing module. Chen et al. [79] combined the dynamic weighing system (WIM) and the cameras along the bridge, proposed a computer-vision-based identification method for the spatial–temporal distribution of vehicle loads on long-span bridges, and conducted field tests and verification on Hangzhou Bay Bridge. In addition, Kim et al. [80] proposed a vision-based method for estimating the cable-stayed forces, which could be used for long-term monitoring under different weather conditions.

Computer-vision-based sensing technology faces three major challenges. Lighting and weather changes lead to unstable image quality, and adaptive exposure, multispectral fusion, and the real-time correction of physical lighting models can be introduced. Occlusion and the limited viewing angle cause information loss, and the blind spot can be filled by multi-camera collaboration, motion prediction frame correction, and 3D reconstruction. With a high compute load and high latency, edge computing hardware, model pruning, and dynamic resolution strategies can reduce energy consumption and speed up. In the future, sensing technology based on computer vision may focus on event-driven sparse sampling and high-accuracy image-processing algorithms to handle environmental interference.

## 3. Discussions on the State-of-the-Art Sensing Technologies

**Figure 1:**
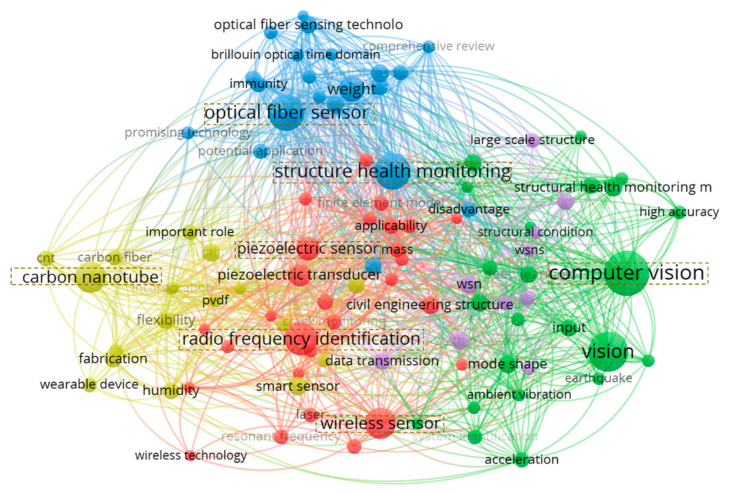
Visualization of sensing technology co-occurrence network.

This research conducted a literature review on structural health monitoring sensing technologies, and provided a visualization of the technology co-occurrence network in the field of structural health monitoring, as shown in Figure 1. It is shown that the terms “radio frequency identification”, “optical fiber sensor”, “carbon nanotube”, “piezoelectric sensor”, “computer vision”, and “wireless sensor” appear with a notably higher frequency in the word cloud, and their font sizes are significantly larger than other keywords, highlighting the pivotal role of these sensing technologies in SHM research.

**Figure 2:**
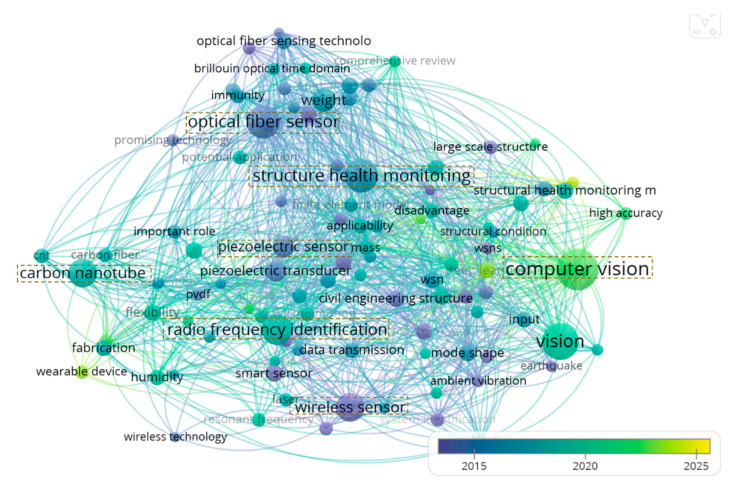
Sensing technology evolution superimposed visualization map.

As shown in Figure 2, this study employs a temporal word cloud visualization to analyze the sensing technological evolution of structural health monitoring from 2014 to 2025. The results demonstrate the following: During the early stage (2014–2016), “wireless sensor”, “optical fiber sensor”, and “piezoelectric sensor” (purple) dominated the SHM field, addressing fundamental monitoring needs and promoting distributed monitoring applications. In the recent phase (2018–2022), “carbon nanotube” and “radio frequency identification” (turquoise) emerged, enabling precise micro-damage detection, while the current period (2022–2025) witnesses the explosive growth of “computer vision” (green), marking SHM’s transition into an intelligent era.

Notably, the technologies across different stages exhibit complementary synergies rather than a simple substitution, exemplified by the combined application of optical fiber and wireless sensing, as well as the cross-modal integration of computer vision with nanomaterials. This evolutionary trajectory clearly reveals SHM’s transformation from wired point monitoring to intelligent, multi-physics fusion monitoring [81,82]. Future research should emphasize the deep integration of emerging technologies (represented by yellow nodes) with conventional solutions (green nodes) to meet the comprehensive monitoring demands for complex infrastructure systems. From a cost–benefit perspective, the sensitivity of carbon nanotubes can reach the single-molecule level, but the cost of high-purity single-walled tubes is still expensive. Although multi-walled tubes are cheap, they sacrifice some performance. Regarding the piezoelectric sensing technology, the unit price of piezoelectric wafers/fiber devices is low, and it can be directly embedded in the structure for self-supply monitoring. However, low-frequency vibration scenarios need to amplify the circuit, increasing the system cost. The use of fiber optic networks requires high upfront expenditures, such as dedicated cable precision connectors and distributed amplifiers [83]. But, once laid, they can provide decades of maintenance-free coverage for long-span bridges, making them economically viable on a large scale. Wireless and computer vision solutions are in the middle: computer vision solutions can utilize inexpensive cameras but incur ongoing costs in computing hardware and lighting control. While wireless sensing technology significantly reduces installation costs when energy-harvesting antennae are feasible, it may waver in environments rich in metals or with scarce electricity.

**Figure 3:**
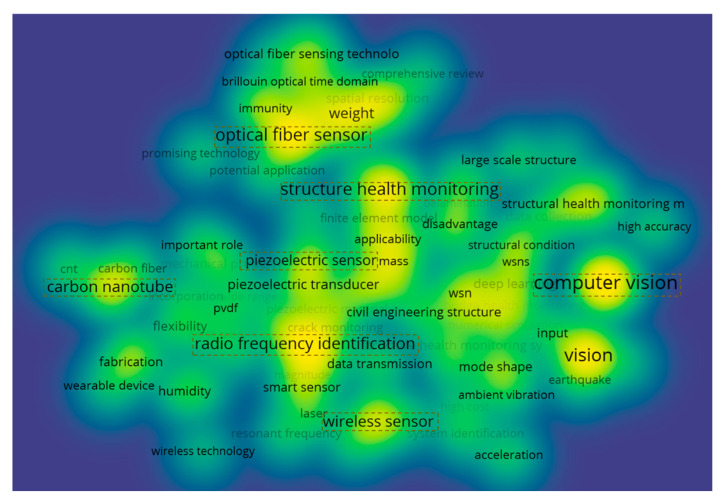
Density distribution map of sensing technology hotspots.

This study systematically analyzes the distribution of technological hotspots in bridge structural health monitoring through density visualization methods, revealing significant gradient characteristics in technology clustering, as shown in Figure 3. “Radio frequency identification” emerges as the most concentrated core research hotspot, with its strong correlation to passive sensing and asset management highlighting its critical role in intelligent operations; “optical fiber sensor” and “computer vision” form the secondary high-density zone, representing precision measurement and intelligent detection approaches, respectively; “carbon nanotube” demonstrates distinctive clustering in flexible sensing, while, among traditional technologies, “wireless sensor” maintains its foundational platform role, and “piezoelectric sensor” continues to deepen its specialization in vibration monitoring. This density distribution pattern not only reveals the evolutionary path from single-parameter monitoring to multi-technology integration, but, more notably, indicates that radio frequency identification technology forms a new density core, suggesting that the deep integration of equipment digital identification and condition monitoring is shaping a new paradigm in SHM. These findings provide important insights for understanding technological transformation trends and planning cutting-edge research directions.

## 4. Conclusions

**Figure 4:**
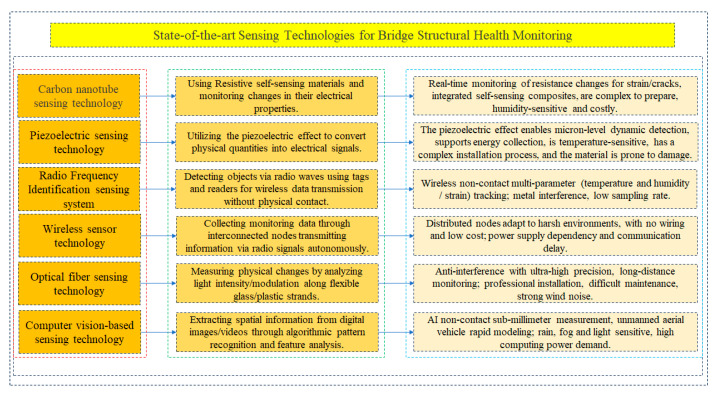
Summary of the main research contents of this study.

In this study, some state-of-the-art sensing technologies, including carbon nanotubes, piezoelectric sensors, RFID systems, wireless sensors, fiber optic sensors, and computer-vision-based sensing technology for bridge SHM have been described in detail (shown in Figure 4), and these sensing technologies can provide high sensitivity, real-time monitoring, and superior anti-interference capabilities, thereby significantly enhancing SHM system performance.

Carbon nanotubes, as a novel high-performance material, exhibit exceptional mechanical properties and electrical conductivity, enabling the real-time detection of strain and cracks, thus substantially improving structural performance and safety. Simultaneously, piezoelectric sensors convert physical quantities into electrical signals, enhancing the reliability of SHM systems by accurately measuring various physical parameters such as the load, effectively monitoring the structural integrity, vibration, and pressure, and providing detailed data on the structural health. RFID sensors offer non-contact identification and automated data capture, enhancing operational efficiency in diverse environments. Their ability to simultaneously detect multiple tags enables rapid inventory management and asset monitoring. Installation and maintenance are also easy due to their wireless design, making them a cost-effective choice for enhanced monitoring and tracking. Wireless sensors, characterized by the absence of complex wiring requirements, offer versatile data collection solutions, facilitating large-scale deployment and real-time data transmission, and can operate stably under diverse environmental conditions, making them widely applicable in SHM. Fiber optic sensors excel in precision and sensitivity, allowing for the accurate monitoring of structural health parameters such as temperature, pressure, and strain. By leveraging fiber optic sensing technology, SHM systems can achieve more detailed data collection and analysis, ensuring the safety and longevity of the infrastructure. Computer-vision-based sensing technology offers non-contact, high-precision data capture, real-time processing, and scalability for diverse applications. It reduces costs through automation, extracts rich spatial and contextual insights, and integrates seamlessly with a machine-learning framework for enhanced decision-making. The integrated application of these advanced sensing technologies significantly enhances the functionality of SHM systems, enabling the comprehensive monitoring of infrastructure health and allowing for the prompt identification of potential risks and hazards, effectively preventing structural failures. Ultimately, the synergistic effect of these sensing technologies greatly improves the safety and lifespan of the infrastructure, providing a solid foundation for societal development.

## Data Availability

The data are available upon request by contacting the corresponding author.
